# A Second Dimension to the Leaf Economics Spectrum Predicts Edaphic Habitat Association in a Tropical Forest

**DOI:** 10.1371/journal.pone.0013163

**Published:** 2010-10-01

**Authors:** Jennifer L. Baltzer, Sean C. Thomas

**Affiliations:** 1 Biology Department, Mount Allison University, Sackville, New Brunswick, Canada; 2 Faculty of Forestry, University of Toronto, Toronto, Ontario, Canada; University Copenhagen, Denmark

## Abstract

**Background:**

Strong patterns of habitat association are frequent among tropical forest trees and contribute to the maintenance of biodiversity. The relation of edaphic differentiation to tradeoffs among leaf functional traits is less clear, but may provide insights into mechanisms of habitat partitioning in these species rich assemblages.

**Methodology/Principal Findings:**

We quantify the leaf economics spectrum (LES) for 16 tree species within a Bornean forest characterized by highly pronounced habitat specialization. Our findings suggest that the primary axis of trait variation in light-limited, lowland tropical forests was identical to the LES and corresponds with the shade tolerance continuum. There was no separation with respect to edaphic variation along this primary axis of trait variation. However, a second orthogonal axis determined largely by foliar P concentrations resulted in a near-perfect separation of species occupying distinct soil types within the forest.

**Conclusions/Significance:**

We suggest that this second axis of leaf trait variation represents a “leaf edaphic habitat spectrum” related to foliar P and potentially other nutrients closely linked to geological substrate, and may generally occur in plant communities characterized by strong edaphic resource gradients.

## Introduction

A central focus of plant ecology is to understand how functional traits contribute to the distribution of species at various spatial scales [1]. Distinct species distribution patterns with respect to abiotic gradients have been documented repeatedly in a wide range of plant communities and play an important role in diversity maintenance. In tropical forests, variation in light, hydrology, and geologic substrate correlate with tree species distributions suggesting a potentially important role of niche partitioning in tropical forest structure and dynamics [2]–[7].

Recently, there has been substantial emphasis on functional traits, particularly foliar traits, in the segregation of species across resource gradients [8]–[12]. The ‘leaf economics spectrum’ [hereafter LES; 13] is a plant strategy axis that forms a continuum of variation from plants having fast returns on investment of nutrients and dry mass (i.e., high physiological rates, nutrient concentrations and low leaf mass per area) to a slow return, stress-tolerant strategy characterized by the opposite set of traits. This spectrum is captured in a single principal component explaining 74% of variation in six key foliar traits within a global data set [GLOPNET; 13].

Similar analyses of smaller, more local datasets have shown variation in the trait relationships that comprise the LES [14]–[17]. When considering this spectrum at the community scale, in the absence of climatic variation, it is reasonable to postulate that the relative importance of traits or the suite of traits contributing strongly to this primary axis of variation may change depending upon the resources that are most limiting in the system in question. Patterns of leaf trait variation may be expected to be most divergent from global patterns where strong gradients of resource availability exist resulting in habitat partitioning among species. For example, within lowland tropical forests, traits comprising the LES should correspond with the shade tolerance continuum that is critical to forest regeneration dynamics [17]–[22]. Soil resources contributing to species distributions also vary among ecosystems and may thus alter the relative importance of foliar traits to the LES. For example, in temperate grassland systems, nitrogen often drives partitioning [23] whereas in tropical systems, soil phosphorus (P) commonly limits plant growth [24]–[26], and can accordingly strongly influence partitioning. P mainly derives from the weathering of soil minerals; consequently, the geologic substrate and degree of weathering determine the availability of P for uptake [27]. P availability differs due to local variation in geology and hydrology and may play a particularly critical role in species partitioning and regional diversity maintenance in lowland tropical forests [4], [8]. P is critical to a variety of plant functions [e.g., RuBP regeneration; 28] and is a major component of key molecules involved in photosynthesis, including phospholipids, nucleic acids, sugar phosphates, and ATP [29]. Foliar P concentrations may thus be expected to contribute more to variation in leaf traits in tropical tree communities characterized by strong P gradients, compared to global relationships.

In the present study, we examine the six foliar traits that comprise the LES [sensu 13] for 16 tree species in a tropical lowland forest within which strong edaphic gradients exist and contribute to striking patterns of habitat association [12], [30]. Our main goals are to: (1) test for the presence of the LES (and other multivariate axes) within a tropical tree community; and (2) test whether species specialized to contrasting edaphic environments separate along these axes in a systematic way. We demonstrate that in lowland tropical forests, the LES describes trait relationships at the local scale and that this primary axis of variation corresponds closely with the shade tolerance continuum. In addition, we show that a second orthogonal axis determined largely by foliar P concentrations results in near-complete separation of tree species occupying distinct habitat types within the forest. We suggest that this ‘leaf edaphic habitat spectrum’ may be more generally applicable in plant communities characterized by strong substrate-based resource gradients.

## Methods

The Sepilok Forest Reserve (5°10′N, 117°56′E; SFR), is a 4294 ha gazetted reserve located in Sabah, Malaysian Borneo. The landscape is topographically variable with alluvial lowlands punctuated by sandstone ridges (30–90 m a.s.l.); this topography results in distinct but adjacent floral assemblages, sandstone hill and lowland dipterocarp forest [31]. Parent material and drainage varies resulting in lower soil water availability and nutrient concentrations and greater light availability on sandstone soils [12], [32], which result in differential plant resource use traits between the two forest types [12]. This pattern is common in the region [33] and is thought to contribute to high tree species diversity in Bornean forests [34].

### Species selection

Four congeneric/confamilial species pairs were selected consisting of one species specialized to the sandstone soil type and one to the alluvium. Four generalists and other common unpaired specialists were also selected to obtain data on a wider range of common species (16 in total). Sapling selection was restricted to healthy individuals with unbroken stems and stem height of 0.5–1.5 m. 15–60 individuals of each species were selected and uniquely tagged. Species information can be found in the online supplement (Table S1). The categorization of species as edaphic generalists or specialists was qualitatively based upon observation with numerous transects walked on each soil type searching for ‘wrongly placed’ individuals (specialists) or species that were fairly common on both sandstone and alluvial soil types (generalists) and corresponds with large plot data for the reserve [35].

### Functional trait data collection

We measured all six traits included in the LES: mass-based photosynthetic and respiration rates (A_mass_ and R_mass_, respectively), leaf mass per area (LMA), leaf N and P concentrations, and leaf lifespan (LL). For gas-exchange measurements, six individuals per species were sampled in high light locations (for generalists, this resulted in 12 sampled individuals; six per soil type). The range of light environments to which ‘high light’ corresponded was 6–13 mol m^−2^ d^−1^. Individual sapling light environment was measured using hemispherical photographs taken directly above the crown using a Nikon Coolpix 900 and FC-E8 fisheye converter (Nikon, Tokyo, Japan). Photographs were analyzed using the program Winscanopy 2001 (Regent Instruments Inc., Québec, Canada). Using a LI-6400 gas-exchange system (Licor, Inc., Lincoln, NE, USA), gas-exchange measurements were made on recent, fully expanded leaves. All measurements were made before noon with cuvette conditions maintained at 350 ppm CO_2_, 60–80% RH and 25–30°C leaf temperature. Measurements were made in 2002 at the beginning of a wet spell. Gas-exchange leaves were harvested, measured for area, dried at 60°C and weighed. Both leaf area and weight excluded the petiole. For compound-leaved species, the measured leaflet was sampled. LMA was calculated and used to convert gas-exchange values to mass-based measures. Leaf tissue, excluding primary and secondary veins, was finely ground and samples wet digested using the sulfuric acid-hydrogen peroxide digest procedure. The digest was analyzed for total N using the phenol blue reaction employing autoanalysis [36] and P by the colorimetric molybdenum blue method [37]. To monitor leaf production, the three most recently expanded leaves on each branch were labeled with non-toxic permanent marker at the base near the petiole every two months from September 2001–March 2002 and again in September and November 2002. Aside from one light-demanding species (*Homolanthus populneus*), there was never an instance when at least one marked leaf could not be found on each branch (for *H. populneus*, LL was conservatively estimated at two months in subsequent analyses). At each census, leaves remaining from the previous census and new leaves produced were tallied. LL was estimated using a demographic approach [38]:
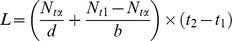
Where *L* is leaf lifespan, *b* is leaf production between censuses, *d* is leaves dropped between censuses, *N_t1_* represents initial number of leaves and *N_tα_* represents number of leaves at *t_1_* plus number of leaves produced between *t_1_* and *t_2_*. This calculation provides an estimate of average leaf longevity based on cumulative leaf production and deaths.

### Additional datasets

To test whether similar patterns exist in other tropical lowland forests, we searched the literature for datasets from other moist tropical forest sites for which comparable data on LES traits as well as quantitative measures of juvenile shade tolerance have been published. We found two sites: La Chonta, Bolivia [18], [39] and Parque Nacional San Lorenzo (hereafter, San Lorenzo), Panama [17]. For the La Chonta dataset, all leaf traits measurements were made on saplings (18) with the exception of foliar P, which was measured on leaves from adult trees (38). The shade tolerance metric used in the Bolivian studies was the Crown Exposure Index (18). For San Lorenzo, we only included the 14 tree species for which shade tolerance was quantified in our analysis. All leaf trait data were collected for adults. The shade tolerance index used in the Panamanian study consisted of factor scores for the first principal component calculated for height growth and proportional survival of first-year seedlings (17).

### Statistical analysis

Analyses followed methods in Wright et al. [13]. Log-10 transformed species trait values were used to ensure normality and homogeneity of variance. A principal components analysis was run on the entire data set to assess whether the LES signal was evident. Generalist species are represented in all analyses twice to quantify shifts in leaf traits between sandstone and alluvial populations. Species' loading scores were then divided by habitat and a Kruskal-Wallis test was used to assess whether habitat type was a good predictor of species' placement along significant axes (loading data were not normally distributed and transformations failed). A regression analysis was run using the primary axis (the ‘LES’) as the predictor of species' whole-plant light compensation point (WPLCP) taken from Baltzer and Thomas [19]. WPLCP is a quantitative measure of shade tolerance in which the X-intercept of the light-growth relationship is estimated for each species and corresponds with a physiologically and ecologically relevant minimum light requirement [19]. To test whether similar patterns exist in other tropical lowland forests, we conducted corresponding analyses for the two additional moist tropical forest sites. Type II regression analysis was used to test whether the slopes differed from unity or the intercepts differed from zero for pairwise relationships of loading values among sites. Differences in these relationships would suggest changes in the relative importance of functional traits among sites. Only five of the six leaf traits were available for San Lorenzo (no R_mass_); to ensure differences in trait loadings were not simply due to this discrepancy we conducted an additional PCA for the Sepilok and La Chonta datasets excluding the R_maas_ data. Results of the corresponding regression anayses can be found in Table S2. Analyses were conducted using R (v. 2.9.0, R Foundation for Statistical Computing).

## Results

**Figure 1:**
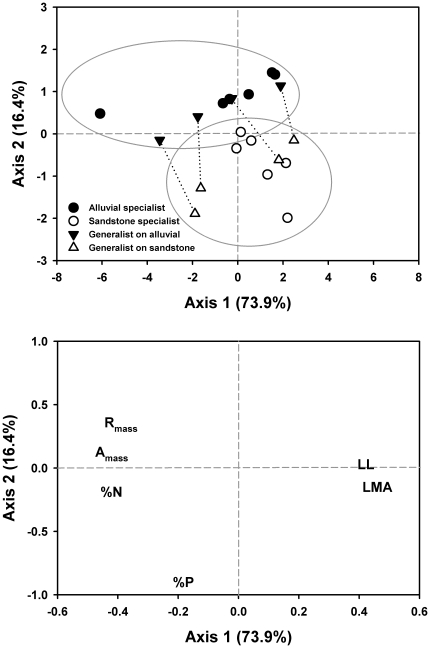
Prinicipal components analysis of the six key leaf economics traits (log-10 transformed). Two significant axes were detected, the first corresponding with the LES with A_mass_, R_mass_, %N and %P loading negatively along the first axis and LMA and LL loading positively (see Table 1 for loading values). The second significant axis corresponds primarily with foliar P concentrations, which load positively with the second axis. Species' loading values are represented on the graph according to the habitat on which they were sampled (closed symbols, alluvial; open symbols, sandstone) and their behaviour as specialists (circles) or generalists (triangles). The two habitat classifications separated significantly along the second axis (Kruskal-Wallis χ^2^ = 13.72, df = 1, P = 0.0002). Generalist species are represented by two data points each: one from the population on the alluvial soils (black triangles) and one from the sandstone soils (white triangles) and connected by a dashed line to demonstrate shifts in loading values between populations.

**Figure 2:**
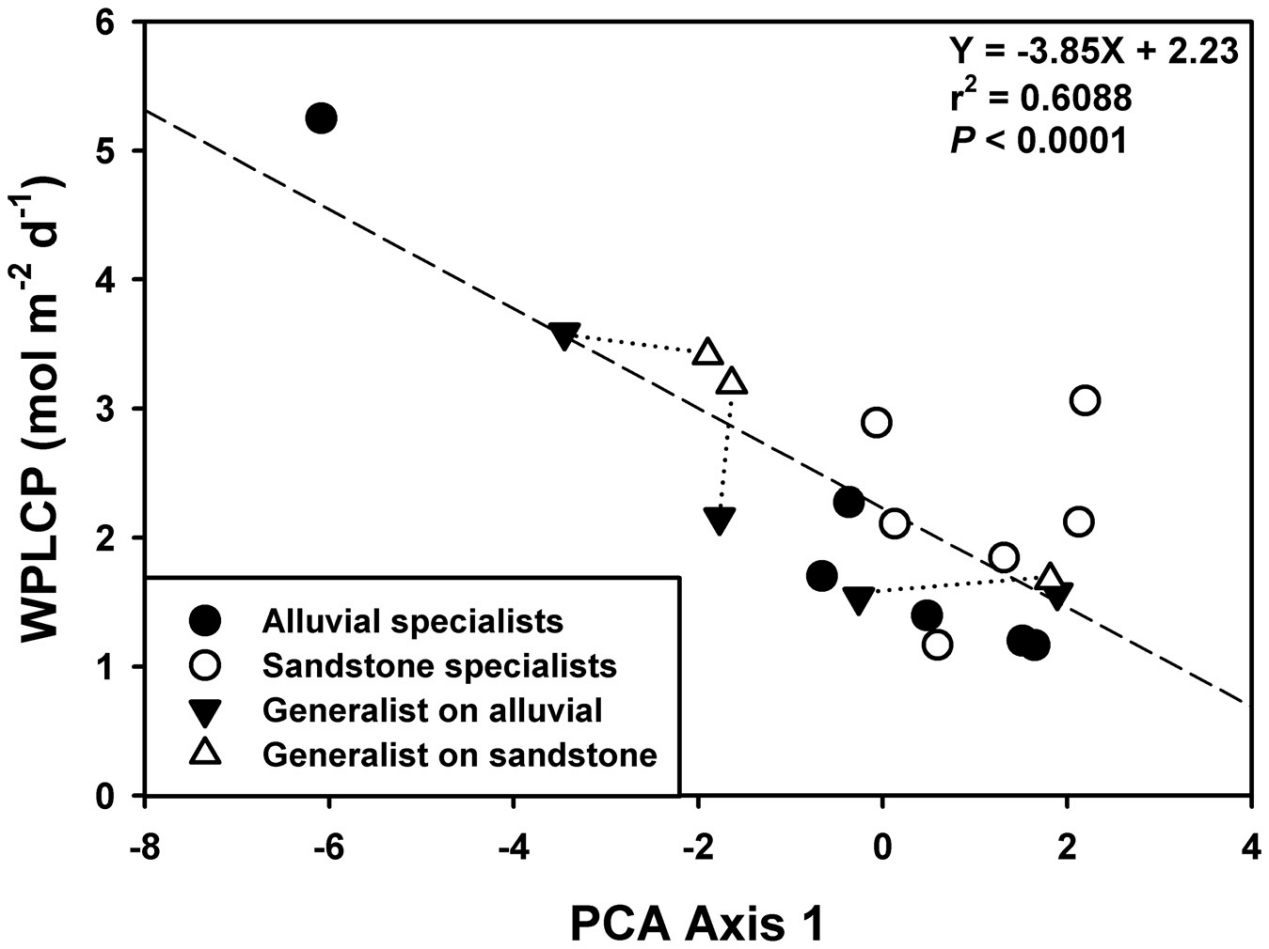
The relationship between the LES and shade tolerance quantified as the whole-plant light compensation point. The LES corresponds with species loadings on PCA axis 1. For a description of the axis see Figure 1 and Table 1. Whole-plant light compensation point values (WPLCP) are from Baltzer and Thomas [19]. Generalist species are represented by two data points each: one from the population on the alluvial soils (black triangles) and one from the sandstone soils (white triangles) and connected by a dashed line to demonstrate shifts in trait values between populations. WPLCP could not be estimated for the sandstone population of *K. laurina* thus only the alluvial population is represented in this figure.

The PCA resulted in two significant axes, explaining 90.3% of the variation in the leaf trait data (Table 1; Fig. 1). The first axis accounted for 73.9% of the variation and corresponded with the LES of Wright et al. (2004). This axis represents a gradient from species with high physiological rates and leaf nutrient concentrations and low LMA and leaf lifespan to species having the opposite traits (Fig. 1). Kruskal-Wallis analysis indicated that the primary axis of variation showed no relationship to habitat preference (χ^2^ = 2.29, df = 1, *P* = 0.1306); however, it correlated with a quantitative shade tolerance metric, WPLCP (Fig. 2). The relationship was strengthened by the high value of one light-demanding species (*H. populneus*) but persisted when this species was excluded (r^2^ = 0.35; *P* = 0.0095). Both La Chonta and San Lorenzo demonstrated identical patterns with the first principal component accounting for 69 and 70% of variation in leaf traits, respectively (Table 1) and significantly predicting juvenile shade tolerance metrics (La Chonta: r^2^ = 0.54; *P*<0.0001; San Lorenzo: r^2^ = 0.39; *P* = 0.0162). All three sites showed stong pairwise correlations for trait scores along the first axis (Table 2).

**Table 1:** Principal components analysis of leaf economics traits for Sepilok (16 species).

Site	Trait	Component 1	Component 2
Sepilok Forest, Malaysia	Variation explained (%)	73.9	16.4
	log A_mass_	−0.457	0.143
	log R_mass_	−0.425	0.345
	log LL	0.450	−0.137
	log LMA	0.418	0.020
	log N	−0.437	−0.187
	log P	−0.204	−0.898
La Chonta, Bolivia	Variation explained (%)	68.5	14.0
	log A_mass_	−0.472	−0.099
	log R_mass_	−0.440	0.172
	log LL	0.403	−0.170
	log LMA	0.442	−0.173
	log N	−0.413	−0.108
	log P	−0.237	−0.943
Parque Nacional San Lorenzo, Panama	Variation explained (%)	69.7	15.9 (ns)
	log A_mass_	0.521	0.243
	log LL	−0.393	−0.714
	log LMA	−0.507	0.057
	log N	0.401	−0.409
	log P	0.412	−0.510

Corresponding analyses for La Chonta (35 species; 18, 38) and San Lorenzo (14 species; 17). All analyses were performed on log-10 transformed variables across species. Two significant components explained a cumulative variation of 90.3% in Sepilok. Variation explained by individual components is provided for each trait. In the corresponding analysis for La Chonta, the second axis was marginally significant (standard deviation of 0.913); combined these two axes explained 82.5% of trait variation. Corresponding analysis using five of six traits (R_mass_ not available) from San Lorenzo; PCA axis 2 was not significant. In all three tables, leaf lifespan and LMA positively correlate with axis 1 while remaining traits are negatively correlated with axis 1. Foliar P concentration loads very strongly onto the second axis compared with all other significant traits in the Malaysian and Bolivian forests but less so in the Panamanian forest.

**Table 2:** Pearson's correlation and regression coefficients (lower CI, upper CI) for pairwise relationships between principal components loadings at the three sites.

	Slope	Intercept	Pearson's ρ	P-value
*Axis 1*				
Sepilok – LaChonta	1.01 (0.92, 1.11)	0.01 (−0.03, 0.05)	0.99	<0.0001
Sepilok – San Lorenzo	0.92 (0.62, 1.36)	0.03 (−0.14, 0.20)	0.98	0.0047
LaChonta – San Lorenzo	0.91 (0.67, 1.22)	0.02 (−0.10, 0.14)	0.99	0.0019
*Axis 2*				
Sepilok – LaChonta	1.08 (0.79, 1.48)	0.08 (−0.06, 0.22)	0.97	0.0011
Sepilok – San Lorenzo	1.01 (0.31, 3.30)	−0.06 (−0.78, 0.66)	0.59	0.2918
LaChonta – San Lorenzo	0.99 (0.29, 3.46)	0.01 (−0.78, 0.79)	0.49	0.3971

The second significant axis for the Sepilok dataset corresponded most closely to variation in leaf P concentration, though all traits with the exception of LMA loaded significantly onto it (Table 1). This second axis resulted in the near-complete separation of species based upon the habitat on which they were sampled (sandstone vs. alluvial; Figure 1; Kruskal-Wallis χ^2^ = 13.72, df = 1, *P* = 0.0002). Only the generalist species *Macaranga hypoleuca*, growing on alluvial soils, showed an axis 2 value lower than that found for the sandstone specialist with the highest axis 2 value, *Sindora coriacea*. The second principal component was virtually identical to Sepilok for La Chonta (Tables 1 & 2); however, the second axis was only marginally significant in La Chonta (SD = 0.91; Table 1). No significant second axis was detected for San Lorenzo, although P does load moderately strongly onto this second axis (Table 1); trait scores for San Lorenzo diverged greatly from those of Sepilok and La Chonta on this axis (Table 2).

## Discussion

### Axis 1: the LES and shade tolerance

The primary axis, which corresponds with the LES of Wright et al. [13] proved to be a good predictor of a quantitative measure of shade tolerance, the whole-plant light compensation point [WPLCP; 19]. This finding is in keeping with previous studies examining LES-related leaf traits and shade tolerance in tropical trees [e.g.], [ 17], [18,20]. Corresponding analyses for La Chonta and San Lorenzo demonstrated that the first principal component was likewise identical to the LES. Correlations between pairs of axis 1 loadings were very high (Table 2). Furthermore, as was the case for the Sepilok dataset, the first component was a significant predictor of available measures of juvenile light requirements for each site. This provides strong evidence for a functional linkage between foliar trait variation comprising the LES and minimum light requirements in tropical forest trees, consistent with the theory that trees that evolved to regenerate in the light-limited environment of a forest understory employ a conservative strategy to ‘pay back’ leaf construction costs through slow turnover rates and long residence times of key nutrients such as nitrogen [13], [21].

At Sepilok, there are differences in the understory light environment associated with soil variation, with the sandstone understory having slightly greater light availability due to the lower basal area and canopy stature [19], [35]. Baltzer and Thomas [19] demonstrated differences between congeneric species pairs in WPLCP with sandstone species having slightly higher light requirements. It is therefore reasonable to hypothesize that there might be some separation of the two groups along the primary axis; however, in spite of the slight difference in WPLCP detected using the phylogenetic framework described above, there were no differences detected in traits associated with the LES (Fig. 1).

### Axis 2: Edaphic specialization and phosphorous

We demonstrate a second significant axis to the LES onto which foliar P loaded most strongly, resulting in near-complete separation of species associated with two distinct edaphic habitats at Sepilok. Sandstone ridges show much lower P availability than the alluvial valleys at this site [32]. Similarly, Paoli [8] demonstrated that foliar P was the strongest predictor of habitat associations in eight closely related *Shorea* species an Indonesian lowland forest. The patterns observed in the present study may thus be broadly applicable to P-limited tropical forests that show strong resource gradients and clearly defined habitat associations.

Initially, we hypothesized that foliar P should play a prominent role in the LES due to P-limitation in tropical lowland forests [25], its critical role in a wide range of physiological functions in plants, and its contribution as a component of molecules such as nucleic acids, phospholipids, ATP and sugar phosphates [29]. If the contribution of foliar P to the LES was primarily through its linkage to photosynthesis, we would expect P to load much more strongly on the primary axis, which it does not; in fact it shows the weakest loading of all traits examined. Previous studies show that the structure of the LES is modified by P via changes in N-A_mass_ relationship, but that P does not contribute directly as a primary determinant of photosynthetic rates, even in P-limited tropical forests [16], [ but see 40].

Why then might P be loading orthogonally to the LES in our analysis? The separation of species associated with sandstone vs. alluvial soils corresponds with a large difference in P availability between the two soil types but total soil N likewise is lower on the sandstone soils [32].

We suggest that the strong loading of foliar P onto the second axis may be due to the availability of interchangeable forms of each nutrient and the relative energetic and carbon costs associated with uptake. There are more interconvertable forms of N than P available to plants. At Sepilok, nitrate availability is lower in sandstone soils while ammonium concentrations are higher [32]. Therefore it may be the case that N is not as strongly limiting as P in this forest, as suggested by high N∶P ratios (15∶1 and higher; data not shown). N uptake is thought to be closely linked to demand whereas P uptake frequently continues even when P is not limiting [41]–[43]. Therefore we expect foliar N to correspond closely with the LES; the proteins necessary to support high photosynthetic and respiration rates have high N demands [44]. With respect to P uptake, plants only have direct access to dissolved phosphate [43], which is replenished by slow diffusion. As the rooting zone becomes devoid of dissolved phosphate, root proliferation or enhanced reliance on mycorrhizal associations and root exudates become necessary to maintain P supply [43]. Root proliferation is considered a primary mechanism for increasing uptake on P-deficient soils whereas on richer soils, up-regulating physiological activity can increase uptake rates [45], [46]; however, root proliferation is costly both in terms of C and N. Recent studies show that in P-deficient soils, the costs of P uptake are very high and rather than maintaining high rates of P uptake, that increased efficiency in resource use is a better strategy [47], [48]. Improved P-use efficiency (i.e., higher cumulative carbon assimilation per unit P investment over the lifetime of the leaf) may be achieved by such means as longer P residence times and preferential allocation of P to photosynthetic tissues (e.g., 46). The clear separation of sandstone vs. alluvial specialists may thus correspond to inherent differences in P use between the two groups that are not directly linked with the LES, but further work is necessary to directly test for such differences.

Sepilok, and Bornean forests more generally, display marked patterns of habitat association driven by edaphic characteristics [30], [33]. A second axis of leaf trait variation might be expected to be strongly expressed under these conditions. Conversely, if species distributions at the local scale are not largely being driven by edaphic limitations, then the contribution of that second axis may disappear. By comparing our findings with LES analyses of La Chonta and San Lorenzo, we can examine these predictions. Loadings of traits onto the second axis were virtually identical for Sepliok and La Chonta. At San Lorenzo, in contrast, all available traits loaded similarly onto the primary axis and no signficant second axis was detected. This qualitatively supports our predictions: soils in Sepilok show the strongest habitat differences in P associated with distinct soils on sandstone ridges and alluvial valleys [32] with correspondingly strong patterns of habitat specificity [35]. La Chonta shows somewhat less pronounced edaphic variability, with anthropogenic dark earths enriched in P [49] that impact soil-vegetation relationships (Peña-Claros et al., submitted). In contrast, recent analyses for Barro Colorado Island, another Panamanian moist lowland forest proximate to San Lorenzo with substantial species overlap, show relatively weak effects of soil P levels on community niche structure [3].

Another interesting feature of the Sepilok analysis is the placement of generalist species along the second axis (Fig. 1). For both sandstone and alluvial habitats, the generalist species occupying those habitats cluster closely with specialists associated with each habitat. This is in keeping with the idea that generalist species are more plastic in their responses to soil resource availability [50] or that some degree of local adaptation has occurred. However, it could also simply be an indication that individuals will take up resources when available (e.g., high P values in generalists occurring on the alluvium) and that sandstone specialists may well be capable of increased P uptake were they to establish and survive on the P-rich alluvial soils. In other words, plasticity could be underlying observed patterns. However, evidence from a reciprocal transplant experiment involving two alluvial and two sandstone specialists at Sepilok suggests that adaptive responses to soil conditions can be pronounced. Although sandstone species showed small increases in foliar P when grown on alluvial soils, foliar P of alluvial specialists was almost double that of the sandstone specialists [32]. When the two groups were grown on sandstone, alluvial specialists maintained higher foliar P concentrations [32] suggesting an important role of differential P-use efficiencies. Whole-plant or cumulative P-use efficiencies may similarly differ among specialists and generalists such that sandstone specialists have a competitive advantage over generalists on P-deficient soils.

In conclusion, our data indicate that the first axis of the leaf economics spectrum (LES) describing patterns of trait covariation in leaf functional ecology corresponds to shade tolerance as quantified by the whole-plant light compensation point, and that this relationship is consistent across tropical lowland forests. However, foliar P is relatively uncorrelated with the first LES axis at sites where soil P strongly influences distribution of vegetation, and instead correlates with a “leaf edaphic habitat spectrum” axis that is quite strong in a tropical tree community characterized by high habitat differentiation among species. Further tests of this leaf edaphic habitat spectrum across substrate-based resource gradients are necessary to assess its generality in tropical forests and other plant communities.

## Supporting Information

Table S1 Study species with families, authorities and habitat on which sampling occurred. Asterisks following habitat type indicate pioneer species. Two habitats were examined in the present study: sandstone-derived ridges having both lower water and nutrient availability (sandstone) and moist, nutrient-rich alluvial valleys (alluvial). Species showing no preference with respect to habitat were classified as generalists and have values on both soil types in the table below; specialists have data for only the habitat on which they are found. A representative voucher specimen for each species is indicated (numbers are the herbarium sheet numbers at the Forest Research Center, Sepilok).
(0.07 MB DOC)

Table S2 Pearson's correlation and regression coefficients (lower CI, upper CI) for pairwise relationships between principal components loadings at the three sites. For these analyses we used only the five traits available for the San Lorenzo dataset: Amass, LMA, leaf lifespan, %N, and %P.
(0.03 MB DOC)
