# Supplementary material for: A Second Dimension to the Leaf Economics Spectrum Predicts Edaphic Habitat Association in a Tropical Forest
Source: PLoS One. 2010 Oct 1;5(10):e13163. doi: 10.1371/journal.pone.0013163 (PMC2948525; doi:10.1371/journal.pone.0013163)
Supplement: Table S1 — Study species with families, authorities and habitat on which sampling occurred. Asterisks following habitat type indicate pioneer species. Two habitats were examined in the present study: sandstone-derived ridges having both lower water and nutrient availability (sandstone) and moist, nutrient-rich alluvial valleys (alluvial). Species showing no preference with respect to habitat were classified as generalists and have values on both soil types in the table below; specialists have data for only the habitat on which they are found. A representative voucher specimen for each species is indicated (numbers are the herbarium sheet numbers at the Forest Research Center, Sepilok). (0.07 MB DOC) [file pone.0013163.s001.doc]

Table S1. Study species with families, authorities and habitat on which sampling occurred. Asterisks following habitat type indicate pioneer species. Two habitats were examined in the present study: sandstone-derived ridges having both lower water and nutrient availability (sandstone) and moist, nutrient-rich alluvial valleys (alluvial). Species showing no preference with respect to habitat were classified as generalists and have values on both soil types in the table below; specialists have data for only the habitat on which they are found. A representative voucher specimen for each species is indicated (numbers are the herbarium sheet numbers at the Forest Research Center, Sepilok).

| **Family** | **Species** | **Habitat** | **Voucher No.** | **PCA 1** | **PCA 2** | **WPLCP** |
| --- | --- | --- | --- | --- | --- | --- |
| Dipterocarpaceae | *Dipterocarpus grandiflorus* (Blco) Blco. | Sandstone | 125511 | -0.0637 | -0.3437 | 2.8882 |
|  | *Dipterocarpus applanatus* V. Slooten | Alluvial | 141410 | -0.6573 | 0.7242 | 1.7036 |
| Dipterocarpaceae | *Shorea multiflora* (Burck) Sym. | Sandstone | 97997 | 2.1966 | -1.9893 | 3.0593 |
|  | *Parashorea tomentella* (Sym.) Meijer | Alluvial | 136262 | -0.3666 | 0.8244 | 2.2737 |
| Ebenaceae | *Diospyros euphlebia* Merr. | Alluvial | 136152 | 1.6463 | 1.403 | 1.1666 |
|  | *Diospyros fusiformis* Kosterm | Sandstone | 141525 | 2.1276 | -0.6916 | 2.1222 |
| Euphorbiaceae | *Mallotus griffithianus* (Muell.Arg) Hook | Sandstone | 99722 | 1.3127 | -0.9621 | 1.8450 |
|  | *Mallotus miquelianus* (Scheff.) Boerl. | Alluvial | 97415 | 0.4813 | 0.9352 | 1.3964 |
| Fabaceae | *Sindora* cf. *coriacea* | Sandstone | - | 0.1298 | 0.0438 | 2.1076 |
| Dipterocarpaceae | *Dipterocarpus caudiferus* Merr. | Alluvial | 102511 | -0.2610 | 0.8395 | 1.5378 |
|  |  | Sandstone |  | 1.8142 | -0.6192 | 1.6699 |
| Euphorbiaceae | *Macaranga hypoleuca* (Rchb.F.&Zoll.)Muell.Arg. | Alluvial* | 91284 | -3.4501 | -0.1476 | 3.5858 |
|  |  | Sandstone* |  | -1.8950 | -1.8922 | 3.4115 |
| Moraceae | *Ficus megaleia* Corner | Alluvial* | 1704 | -1.7688 | 0.4111 | 2.1574 |
|  |  | Sandstone* |  | -1.6384 | -1.2885 | 3.1866 |
| Myristicaceae | *Knema laurina* (Blume) Warb. | Alluvial | 102512 | 1.8917 | 1.1380 | 1.5747 |
|  |  | Sandstone |  | 2.4783 | -0.1555 | nd |
| Euphorbiaceae | *Homolanthus populneus* (Giesl.) Pax. | Alluvial* | 129402 | -6.0849 | 0.4786 | 5.2500 |
| Lauraceae | *Eusideroxylon zwageri* Teijsm. Et. Binn. | Alluvial | 69481 | 1.5151 | 1.4504 | 1.2007 |
| Malvaceae | Scaphium affine Pierre | Sandstone | 119196 | 0.5926 | -0.1585 | 1.1677 |
