# Supplementary material for: A Second Dimension to the Leaf Economics Spectrum Predicts Edaphic Habitat Association in a Tropical Forest
Source: PLoS One. 2010 Oct 1;5(10):e13163. doi: 10.1371/journal.pone.0013163 (PMC2948525; doi:10.1371/journal.pone.0013163)
Supplement: Table S2 — Pearson's correlation and regression coefficients (lower CI, upper CI) for pairwise relationships between principal components loadings at the three sites. For these analyses we used only the five traits available for the San Lorenzo dataset: Amass, LMA, leaf lifespan, %N, and %P. (0.03 MB DOC) [file pone.0013163.s002.doc]

Table S2. Pearson’s correlation and regression coefficients (lower CI, upper CI) for pairwise relationships between principal components loadings at the three sites. For these analyses we used only the five traits available for the San Lorenzo dataset: A_mass_, LMA, leaf lifespan, %N, and %P.

|  | **Slope** | **Intercept** | **Pearson’s ρ** | **P-value** |
| --- | --- | --- | --- | --- |
| *Axis 1* |  |  |  |  |
| Sepilok – LaChonta | 1.00 (0.89, 1.12) | 0.00 (-0.05, 0.06) | 0.98 | 0.0001 |
| Sepilok – San Lorenzo | 0.99 (0.70, 1.41) | 0.03 (-0.13, 0.18) | 0.98 | 0.0031 |
| LaChonta – San Lorenzo | 0.99 (0.75, 1.30) | 0.02 (-0.10, 0.14) | 0.99 | 0.0143 |
| *Axis 2* |  |  |  |  |
| Sepilok – LaChonta | 0.92 (0.73, 1.18) | 0.05 (-0.04, 0.16) | 0.99 | 0.0010 |
| Sepilok – San Lorenzo | 0.93 (0.30, 2.89) | 0.06 (-0.56, 0.68) | 0.65 | 0.2341 |
| LaChonta – San Lorenzo | 1.00 (0.30, 3.35) | 0.00 (-0.74, 0.74) | 0.55 | 0.3289 |
